# Thrombin-derived host defence peptide modulates neutrophil rolling and migration *in vitro* and functional response *in vivo*

**DOI:** 10.1038/s41598-017-11464-x

**Published:** 2017-09-11

**Authors:** Chun Hwee Lim, Manoj Puthia, Marta Butrym, Hui Min Tay, Michelle Zi Yi Lee, Han Wei Hou, Artur Schmidtchen

**Affiliations:** 1 Lee Kong Chian School of Medicine, Nanyang Technological University, Singapore, Singapore; 2 NTU Institute for Health Technologies, Interdisciplinary Graduate School, Nanyang Technological University, Singapore, Singapore; 3 Division of Dermatology and Venereology, Department of Clinical Sciences, Lund University, Lund, Sweden; 4 School of Chemical and Life Sciences, Singapore Polytechnic, Singapore, Singapore

## Abstract

Host defence peptides (HDPs) derived from the C-terminus of thrombin are proteolytically generated by enzymes released during inflammation and wounding. In this work, we studied the effects of the prototypic peptide GKY25 (GKYGFYTHVFRLKKWIQKVIDQFGE), on neutrophil functions. *In vitro*, GKY25 was shown to decrease LPS-induced neutrophil activation. In addition, the peptide induced CD62L shedding on neutrophils without inducing their activation. Correspondingly, GKY25-treated neutrophils showed reduced attachment and rolling behaviour on surfaces coated with the CD62L ligand E-selectin. The GKY25-treated neutrophils also displayed a dampened chemotactic response against the chemokine IL-8. Furthermore, *in vivo*, mice treated with GKY25 exhibited a reduced local ROS response against LPS. Taken together, our results show that GKY25 can modulate neutrophil functions *in vitro* and *in vivo*.

## Introduction

Antimicrobial peptides, also known as host defence peptides (HDPs), play important roles in innate defence in skin and during wounding^1^. They are mostly cationic and amphipathic molecules with direct and rapid antimicrobial actions against a broad-spectrum of microbes including both Gram-negative and Gram-positive bacteria, viruses, as well as fungi^1–3^. Lately, HDPs have attracted significant therapeutic interest, not only due to their antimicrobial effects, but also their ability to modulate host immune responses^1, 4–6^.

Recently, novel HDPs derived from the C-terminus of thrombin (thrombin C-terminal peptides, TCPs) have been described. These TCPs exhibit physicochemical properties similar to classical HDPs – including cationicity, α-helicity and amphipathicity^7, 8^. Further investigations reveal the antibacterial and anti-endotoxic properties of these TCPs^7^. Importantly, these TCPs can be generated by proteolytic cleavage *in vitro* ^7^, and are detected in human wound fluids *in vivo* ^9^. Using a prototypic TCP, GKY25 (GKYGFYTHVFRLKKWIQKVIDQFGE) for the treatment of mice challenged with lipopolysaccharides (LPS) or *Pseudomonas aeruginosa*, it was shown that the peptide improved animal survival^7, 10^. More recently, it was also demonstrated that GKY25 inhibits monocyte and macrophage responses to LPS via direct LPS and cell interactions^11^.

Neutrophils, as the most abundant leukocytes in the blood, are among the first cells that respond to injury or inflammation. Their primary actions include extravasation and migration toward the site of injury followed by microbial killing through different mechanisms, including the generation of reactive oxygen species (ROS), formation of neutrophil extracellular traps (NETs) and release of HDPs^12^. Some HDPs have been shown to modulate neutrophil functions; for example, the human cathelicidin LL37, produced by neutrophils, keratinocytes and other cell-types, is a chemo-attractant for neutrophils, facilitating their activation, degranulation, production of defensins, ROS generation and NET formation^13–15^. This prompted us to investigate the effects of TCPs in modulating neutrophil functions. In this work, we show that the prototypic peptide GKY25 reduces neutrophil rolling and chemotactic responses through the shedding of CD62L. We also demonstrate the efficacy of GKY25 in reducing neutrophil-derived ROS response against LPS *in vivo*. Taken together, our results show for the first time that GKY25 can modulate neutrophil responses *in vitro* and *in vivo*.

## Methods

### Materials

Polymorphoprep was purchased from Axis-Shield, Scotland. Unlabelled and tetramethylrhodamine (TAMRA)-labelled GKY25 (GKYGFYTHVFRLKKWIQKVIDQFGE) and IVE25 (IVEGSDAEIGMSPWQVMLFRKSPQE), were ordered from Biopeptide, USA. The fluorescently-labelled antibodies Pacific Blue (PB)- or Brilliant Violet 510 (BV510)-anti-CD45 (clone HI30), fluorescein isothiocyanate (FITC)-anti-CD66b (clone G10F5), allophycocyanin (APC)-anti-CD11b (clone ICRF44), phycoerythrin (PE)/Cy7-anti-CD62L (clone DREG-56), PE-anti-P selectin glycoprotein ligand (PSGL)-1 (clone KPL-1), PE/Cy5-anti-CXCR1 (clone 8F1) and peridinin chlorophyll protein complex (PerCP)/Cy5.5-anti-CXCR2 (clone 5E8) were from BioLegend, USA. The rat monoclonal anti-neutrophil antibody (NIMP-R14) was from Abcam, USA. Alexa-Fluor (AF)-568 anti-rat IgG was from Life Technologies, USA. The live-dead stain 4′6-diamidino-2-phenylindole (DAPI) and intracellular ROS fluorescent probe 2′,7′-dichlorodihydrofluorescein diacetate (H_2_DCFDA) were bought from Thermo-Fisher Scientific, USA. GM6001 was purchased from Millipore, USA. Lactate dehydrogenase (LDH) cytotoxicity kit was purchased from Pierce, USA. Luminol, horseradish peroxidase (HRP), calcium chloride (CaCl_2_), LPS from *Escherichia coli* O111:B4 and phorbol 12-myristate 13-acetate (PMA) were bought from Sigma-Aldrich, USA. ELISA for soluble CD62L (sCD62L) was from RayBiotech, USA. E-selectin used for rolling assay was from Peprotech, USA. The 96-well transwell plates with 3.0 µm inserts were from Corning, USA. The human chemokine interleukin-8 (IL8) was from Miltenyi Biotech, Germany. The ROS probe L-012 was obtained from Wako Chemicals, Germany. The Optimum Cutting Temperature (O.C.T.) compound was bought from VWR, USA.

### Ethics statement

Human whole blood samples were collected with written informed consent obtained from each human subject who participated. All methods involving the use of human blood samples, including the blood collection process, were performed in accordance with the guidelines and regulations as reviewed and approved by the Nanyang Technological University Institutional Review Board, Singapore (IRB-2014-10-041). The animal models were approved by the Laboratory Animal Ethics Committee of Malmö/Lund (permit numbers M89-14 and M88-14) and experiments were conducted in accordance with the guidelines of the Swedish Animal Welfare Act SFS 1988:534.

### Isolation of polymorphonucleated cells (PMNs)

Neutrophils, or PMNs, were isolated from whole blood anti-coagulated with sodium citrate. Briefly, approximately 2 parts of blood were layered on 1 part of Polymorphprep before centrifugation at 500x *g*, 23 °C for 30 min (without brakes). The layer containing PMNs was harvested before erythrocyte lysis with Erythrocyte Lysis Buffer (eBioscience, USA). PMNs were then re-suspended in RPMI without phenol red supplemented with 10% foetal bovine serum (FBS). Flow cytometry was then performed on either LSR-II or LSR-Fortessa-X20 (Becton-Dickinson, USA) and the analyses were performed using Flowjo (Treestar, USA). PMNs were defined as CD45^+^CD66b^+^ cells and percentage of CD66b^+^ cells over live CD45^+^ was determined to be >95%, unless otherwise stated, before the start of experiments.

### Peptide uptake by PMNs

Uptake of IVE25 or GKY25 was assessed with flow cytometry and confocal microscopy. PMNs (5 × 10^5^ cells/ml) were incubated with 5 µM of TAMRA-labelled GKY25 (T-GKY25) or (T-IVE25) for the indicated periods of time at 37 °C. PMNs were then stained with FITC-anti-CD66b and 10 µm DAPI in FACS buffer containing phosphate-buffered saline (PBS) supplemented with 5% foetal bovine serum (FBS) before flow cytometry using either the LSR-II or LSR-Fortessa-X20. Median fluorescence intensities (MFIs) of TAMRA were determined from live, CD66b^+^ cells for analyses.

For the visualisation of peptide uptake, 200,000 total cells were first washed with Tris buffer (TBS), fixed with 4% paraformaldehyde and washed again with TBS-T (0.1% Triton-X in TBS). Then, PMNs were re-suspended in TBS-T and centrifuged onto 0.1 µg/ml poly-L-lysine coated coverslips in a 24-well plate. Finally, the coverslips were mounted onto the DAPI-containing mounting medium Fluoroshield (Thermo-Scientific, USA) and imaged with the LSM 800 with Airyscan confocal microscope (Zeiss, Germany).

### Lactate dehydrogenase (LDH) cytotoxicity assay

Cytotoxic effects of TCPs were assessed with the LDH release assay. PMNs were first treated with the indicated concentrations of IVE25 or GKY25 for 1 hr at 37 °C on a 96-well flat-bottom plate. The supernatants were then used for LDH assay according to manufacturer’s instructions. Absorbance readings at 490 and 680 nm were acquired using the Cytation 3 Cell-Imaging Multi-Mode reader (Research Instruments, USA). LDH activity was determined by subtracting absorbance reading at 680 nm from the reading at 490 nm (A_490_ − A_680_). Per cent cytotoxicity was calculated as below:

1 $$ \% \mathrm{Cytotoxicity}=\frac{\mathrm{Compound}\,\mathrm{treated}\,\mathrm{LDH}\,\mathrm{activity}-\mathrm{Spontaneous}\,\mathrm{LDH}\,\mathrm{activity}}{\mathrm{Maximum}\,\mathrm{LDH}\,\mathrm{activity}-\mathrm{Spontaneous}\,\mathrm{LDH}\,\mathrm{activity}}\times 100$$

### Assessment of PMN surface markers

PMN surface markers were assessed by flow cytometry. Isolated PMNs (5 × 10^5^ cells/ml) were treated with 5 µM of IVE25 or GKY25, 10 ng/ml LPS or 12.5 ng/ml IL8 as indicated for 1 hr at 37 °C with periodic light shaking. PMNs (1 × 10^6^ cells/ml) pre-incubated in 1:25 ratio by volume of H_2_O, DMSO or 100 μM GM6001 for 30 min at 37 °C were also similarly treated, as indicated, at a final cell density of 5 × 10^5^ cells/ml. To assess PMN activation, cells were stained with 1:200 dilution of FITC-anti-CD66b, APC-anti-CD11b, PE/Cy7-anti-CD62L and PE-anti-PSGL1 with 10 µM of DAPI diluted in FACS buffer. For analysis of expression of CXCR1 and CXCR2, treated PMNs were stained with 1:200 dilution of FITC-anti-CD66b and 1:100 dilution of PE/Cy5-anti-CXCR1 or PerCP/Cy5.5-anti-CXCR2 with 10 µM DAPI diluted in FACS buffer. For whole blood PMN assessment, blood was treated with different concentrations of IVE25 or GKY25 (as indicated) in the presence or absence of 10 ng/ml LPS for 2 hrs at 37 °C with periodic light shaking. Erythrocytes were then lysed with Erythrocyte Lysis Buffer before staining with the respective fluorescently-labelled antibodies and DAPI diluted in FACS buffer. Finally, flow cytometry was performed using the LSR-II or LSR-Fortessa-X20. The MFIs of the respective markers were determined from live, CD66b^+^ cells for statistical analyses.

### Luminol-based ROS measurement

PMNs’ ROS generation was assessed based on luminol chemiluminescence. ROS assay medium containing 100 µM luminol and 2.4 U/ml HRP in RPMI with 10% FBS was first prepared and used as the dilution medium for all treatment conditions. Final working concentration was 50 µM luminol and 1.2 U/ml HRP after the addition of treatment-containing ROS assay medium to the cell suspension. The assay was performed in a white, flat bottom 96-well plate.

First, PMNs (1 × 10^6^ cells/ml) were pre-warmed to 37 °C for 30 min. Next, the PMN suspension was added to the ROS assay medium containing either 50 nM PMA (positive control), 20 ng/ml LPS, 10 µM IVE25 or 10 µM in 1:1 ratio by volume. For the pre-treatment of PMNs, PMNs (5 × 10^5^ cells/ml) were first treated with 10 ng/ml LPS, 5 µM IVE25 or 5 µM GKY25 for 30 min at 37 °C, washed, re-suspended to 1 × 10^6^ cells/ml then added to ROS assay medium containing 20 ng/ml LPS in 1:1 ratio by volume. Chemiluminescence was read immediately with the Cytation 3 Cell-Imaging Multi-Mode reader every 3 min for 3 hrs in 37 °C. Area under the curve (AUC) was then determined from the chemiluminescence plot over time for statistical analyses.

### Flow cytometry-based ROS assessment

PMNs’ ROS generation was also assessed using flow cytometry. PMNs (1 × 10^6^ cells/ml) were pre-incubated with 10 μM H_2_DCFDA for 30 min at 37 °C before co-treatment with 5 μM IVE25 or 5 μM GKY25 and 10 ng/ml LPS for 1 hr at 37 °C. PMNs treated with 25 nM PMA was used as positive control. Cells were then stained with APC-anti-CD11b (Suppl. Figure 2B), PE/Cy7-anti-CD62L (Suppl. Figure 2C) and DAPI. Flow cytometry was then performed on the LSR-Fortessa-X20. As H_2_DCFDA is excited and emits fluorescence at the same wavelengths as FITC, a separate sample without H_2_DCFDA was stained with FITC-anti-CD66b to determine the PMN population in the forward scatter against side scatter plot. MFIs for H_2_DCFDA were determined and used for statistical analyses.

### Enzyme-linked immune-sorbent assay (ELISA) for soluble CD62L

To assess whether CD62L is shed, supernatants were collected from PMNs (5 × 10^5^ cells/ml) treated with 5 μM of GKY25 for 1 hr. The supernatants were then used for ELISA to measure sCD62L levels following manufacturer’s instructions, while the cells were subjected to flow cytometry as described to assess cell surface CD62L levels. Absorbance readings for ELISA were acquired using the Cytation 3 Cell-Imaging Multi-Mode reader.

### PMN rolling characterisation

PMN rolling was studied using a microfluidic polydimethylsiloxane (PDMS) straight-channel microdevice (1 cm length by 400 µm width by 60 µm height) previously reported by Hou *et al*. (2016). Briefly, the PDMS microdevice was pre-coated with 50 μg/ml E-selectin for 1 hr at 4 °C before blocking with 0.5% bovine serum albumin (BSA) in PBS for 30 min at room temperature. For the assay, PMNs (1 × 10^6^ cells/ml) were first treated with 5 μM of IVE25 or GKY25 for 1 hr at 37 °C. Then, 20 μM of CaCl_2_ was added to facilitate the cells’ binding and rolling on the E-selectin functionalised channel. Phase contrast images were captured at 0.5 s intervals for 30 s with 20x magnification using the MetaMorph software (Molecular Devices).

The rolling velocity of individual cell was determined with MATLAB (Mathworks®). Briefly, image contrast was adjusted and contrast thresholding was applied for cell segmentation and background elimination. Cells which appeared on less than 10 frames were discarded. Then, blob analysis was applied to calculate approximate cell size and centroid position from segmented regions using 8-connected pixels criterion to eliminate non-cell components. The centroid positions of the segmented cells were compared with consecutive frames using K-nearest neighbourhood to determine the rolling distance, which was used to calculate rolling velocity by dividing the distance by sampling time (0.5 s)^16^.

### Transwell migration assay

PMN chemotactic function was assessed using 96-well transwell plates with 3.0 µm pore inserts. PMNs (5 × 10^5^ cells/ml) were treated with 12.5 ng/ml IL8 or 5 µM of either IVE25 or GKY25 for 1 hr at 37 °C. The cells were then washed and reconstituted to 1 × 10^6^ cells/ml. Then, 75 µl of the cell suspension was added to the respective upper chambers, with the lower chambers containing 235 µl of 12.5 ng/ml IL8. Spontaneous migration control was included with the bottom chamber containing only culture medium. PMNs were then allowed to migrate to the lower chamber for 2 hrs at 37 °C. Next, 200 µl of cells from the lower chamber was stained with 2 µg/ml Hoechst 33342 in a 96-well plate and the cells were counted with the INCell Analyzer 2200 (GE Healthcare, USA). In addition, 200 μl of washed cells from each condition was stained and counted to represent complete migration. Per cent of migrated cells against total cells was determined. The assay was performed 11 times with PMNs isolated from 6 individuals. Each assay was normalised against its positive control (untreated PMNs migration against IL8) and the results were pooled for statistical analysis.

2 $$ \% Migration=\frac{Migrated\,cells}{Total\,cell\,count}\times 100 \% $$

### ROS assessment *in vivo*

Male BALB/c mice between 8–10 weeks old were used. The back of the mice was shaved for imaging purpose. Mice were pre-treated with 100 μg GKY25 (1 mg/ml in deionised water) intraperitoneally (i.p.). Fifteen minutes after GKY25 treatment, 100 μg LPS (1 mg/ml in deionised water) was injected subcutaneously (s.c.) in the scruff of the neck. Fifty minutes after LPS injection, the ROS probe L-012 was injected i.p. (100 μl, 5 mg/ml). The presence of ROS in the mice was imaged 10 min after L-012 injection using an IVIS SPECTRUM/200 Imaging System (Caliper Life Sciences, USA). Data acquisition and analyses were performed using Living Image version 4.4 (Caliper Life Sciences). Results are expressed as total flux (photons/second).

### Neutrophil infiltration *in vivo*

Male BALB/c mice between 8–10 weeks old were used. In the first setup, mice were pre-treated with 100 μg GKY25 (1 mg/ml in deionised water) i.p. as above. Fifteen minutes after GKY25 treatment, 100 μg LPS (1 mg/ml in deionised water) was injected s.c. in the scruff of the neck. In the second experimental setup, mice were injected subcutaneously with 5 μg LPS in the absence or presence of 100 μg GKY25. In both cases, the mice were sacrificed after 1 hr, after which skin tissues from the LPS-injection site were harvested and embedded in O.C.T. compound. Then, 10 μm cryo-sections were obtained and fixed in in cold acetone-methanol (1:1 v/v). After fixing, sections were blocked with 1% BSA and stained with anti-neutrophil antibody (NIMP-R14) followed by secondary staining with AF568 anti-rat IgG. Nuclei were then stained with 50 μM DAPI. Finally, images were taken using the Olympus Optical AX60 fluorescence microscope (Olympus, USA). Four random microscopic views from each stained section were scored independently by 2 observers on a scale of 0–10 for neutrophil infiltration, where 0 is no neutrophil infiltration and 10 is maximum neutrophil infiltration.

### Statistical analyses

Statistical analyses were performed on Prism version 6.0 (GraphPad, USA). The respective statistical tests performed were as indicated. Results were presented in Mean (SD). Statistical significance was represented in the form of *p* values as such: *p < 0.05, **p < 0.01, *** *p* < 0.001 or ****p < 0.0001.

### Disclosure

A.S. is a founder of in2cure AB, a company developing peptides for human therapy. The peptide GKY25 is patent protected.

## Results

### GKY25 is selectively taken up by PMNs

**Figure 1:**
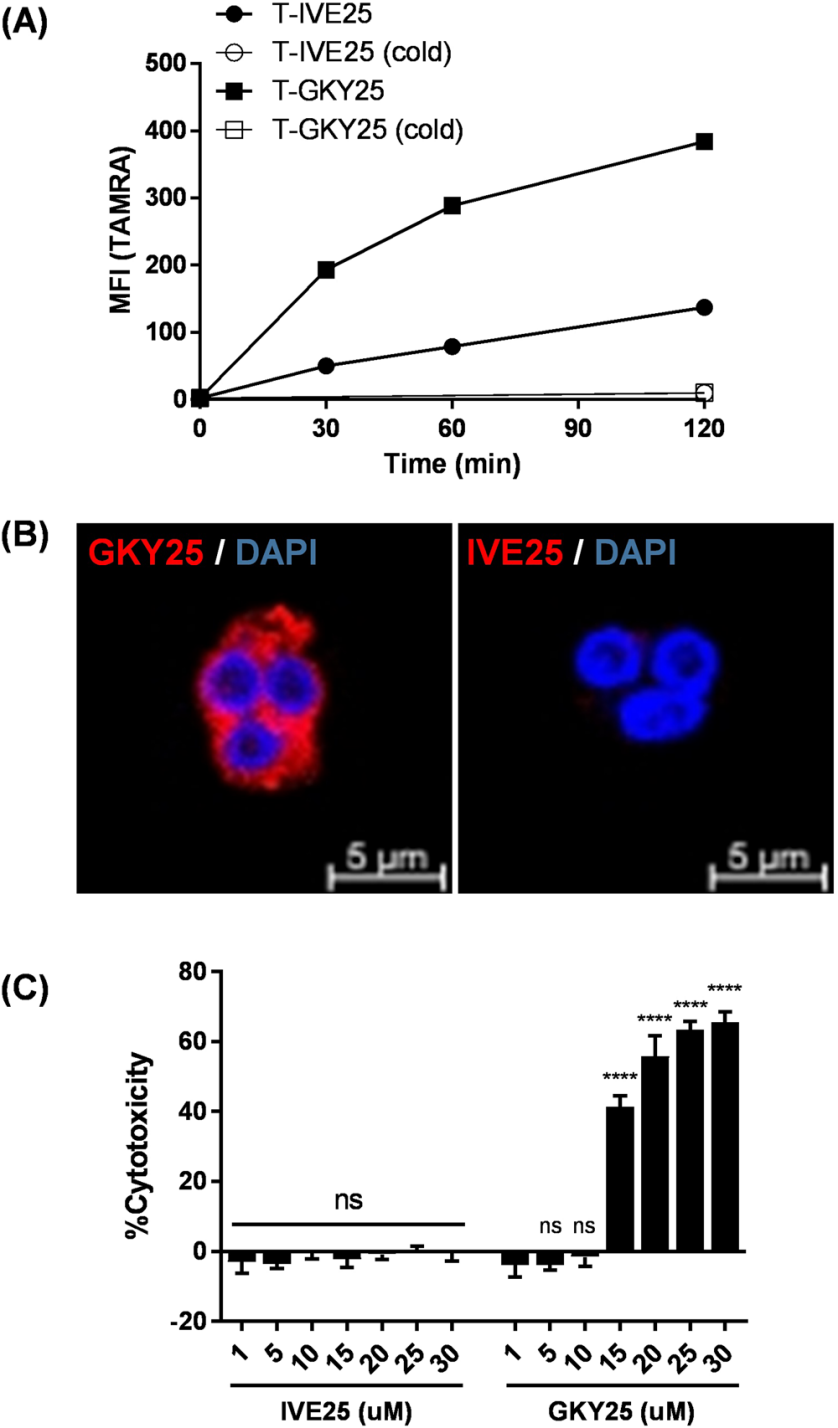
Uptake of GKY25 by PMNs and analysis of LDH release. Uptake of GKY25 was assessed by (**A**) flow cytometry and (**B**) confocal microscopy. (**A**) PMNs (5 × 10^5^ cells/ml) were incubated with 5 µM T-IVE25 or T-GKY25 for 0, 30, 60, 120 min at 37 °C or on ice for 120 min before flow cytometry (n = 3). (**B**) PMNs (5 × 10^5^ cells/ml, 200,000 cells in total) were incubated with 5 µM T-IVE25 or T-GKY25 for 120 min before confocal imaging. (**C**) PMNs incubated with 1, 5, 10, 15, 20, 25 or 30 µM IVE25 or GKY25 for 1 hr were assessed for cytotoxicity using the LDH release assay. (−) Baseline. One-way ANOVA. Figures are representative of 2 (**A**,**B**) or 3 (**C**) independent experiments.

As a first step to elucidate possible immunomodulatory effects of GKY25 on PMNs, we studied the peptide-PMN interaction using flow cytometry and confocal microscopy. Flow cytometry analysis demonstrated a time-dependent uptake of TAMRA-labelled GKY25 (T-GKY25) by PMNs (Fig. 1A). Although it was observed that the MFI for T-IVE25 increased slightly over time, uptake of T-GKY25 was significantly higher, demonstrating a more selective uptake of GKY25. The uptake of T-GKY25 was further confirmed by confocal microscopy, where only T-GKY25 and not T-IVE25 was detected in the cytoplasm of the cells (Fig. 1B).

HDPs have been reported to be cytotoxic to mammalian cells^17^, therefore we set out to examine the potential cell permeabilising effects of GKY25. The results show that 1 hr treatment with GKY25 was non-cytotoxic at doses up to 10 µM and displayed dose-dependent cytotoxicity from 15–30 μM (Fig. 1C). GKY25 at 5 µM was also non-cytotoxic for up to 4 hrs (Supp. Figure 1A). In addition, treatment with 5 µM of GKY25 for 1 hr did not significantly reduce PMNs ability to generate ROS when compared to LPS-treated PMNs against PMA, a strong chemical stimulator for ROS generation (Supp. Figure 1B), thus indicating that PMNs remained functionally active at this level of GKY25 *in vitro*. Taken together, the results indicate that GKY25 is taken up by PMNs, is non-cytotoxic at doses at 10 µM or below, and that it does not affect the innate functional responses of PMNs.

### GKY25 inhibits LPS-induced PMN activation

**Figure 2:**
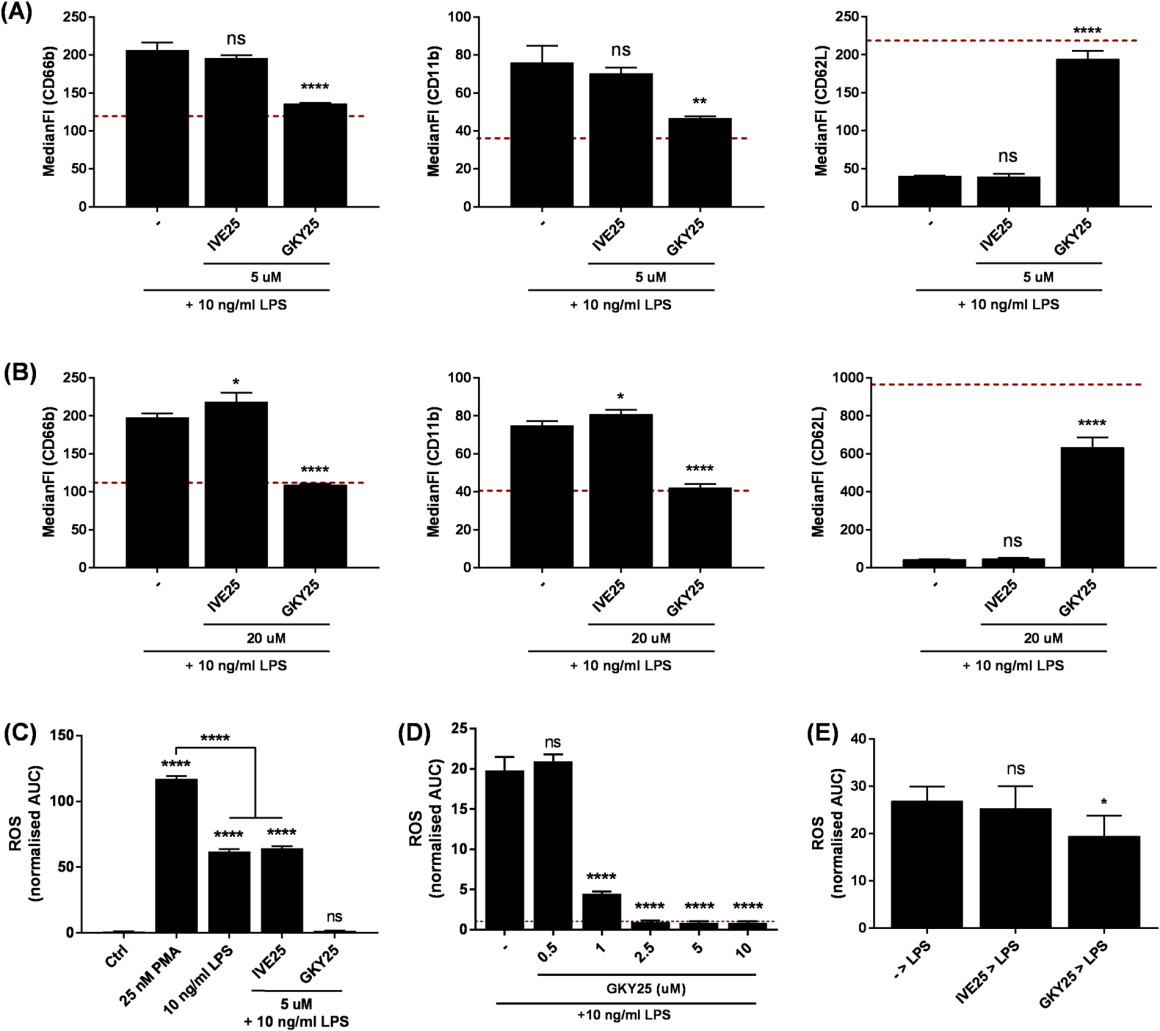
Effects of GKY25 on LPS responses of PMNs. PMN activation markers were assessed by flow cytometry on (**A**) isolated PMNS (5 × 10^5^ cells/ml) co-treated with either 5 µM IVE25 or 5 µM GKY25 and 10 ng/ml LPS for 1 hr at 37 °C (n = 3) and (**B**) PMNs in whole blood co-treated with either 20 µM IVE25 or 20 µM GKY25 and 10 ng/ml LPS for 2 hrs at 37 °C (n = 3). PMNs stimulatory functions were assessed using the luminol-based ROS measurement – pre-warmed isolated PMNs (5 × 10^5^ cells/ml) were co-treated with (**C**) either 5 µM IVE25 or 5 μM GKY25 and 10 ng/ml LPS (n = 5) and (**D**) 0.5, 1.0, 2.5, 5.0 or 10 µM GKY25 and 10 ng/ml LPS (n = 5). For positive control, PMNs in (**C**) were also treated with 25 nM PMA. (**E**) PMNs (5 × 10^5^ cells/ml) pre-treated with 5 µM IVE25 (*IVE25* > *LPS*) or 5 μM GKY25 (*GKY25* > *LPS*) were also subjected ROS induction with 10 ng/ml LPS (n = 5). ROS measurements were done every 3 min for 3 hrs at 37 °C and AUCs were determined for statistical analyses. (−) Baseline. One-way ANOVA. Figures are representative of 3 independent experiments.

Previous studies have shown that TCPs, including GKY25, can bind to both LPS and monocytes/macrophages and inhibit endotoxic effects in cell models *in vitro* as well as in mouse models *in vivo* ^7, 10, 11^. Here, we explored the potential anti-endotoxic effects of GKY25 on neutrophils. First, the expression levels of activation markers’ on PMNs in response to LPS with or without added GKY25 or the control peptide IVE25 were assessed. Three surface markers were selected to assess neutrophil activation - CD66b, which is upregulated as a degranulation marker^18^, CD11b which mediates neutrophil adhesion during activation, and CD62L which is shed upon neutrophil activation^19, 20^. As expected, LPS induced upregulation of CD66b and CD11b with a concomitant downregulation of CD62L (Fig. 2A). In the presence of GKY25 however, the LPS-mediated change in the expression levels of these activation markers was significantly inhibited, with levels close to those found in untreated cells (baseline). The control peptide IVE25 did not show these inhibitory effects (Fig. 2A). Next, we examined the changes in PMN markers in whole blood in response to LPS in the presence or absence of GKY25. Similarly, while LPS alone induced significant changes to the activation markers, GKY25 but not IVE25 abolished the effect (Fig. 2B).

As LPS has been reported to induce a low, but still detectable, ROS response in human PMNs *in vitro* ^21–24^, we next decided to evaluate the effects of GKY25 on LPS-treated PMNs. LPS treatment of PMNs yielded an increase in ROS levels, albeit to a lesser extent as compared to PMA-treated cells (Fig. 2C and Suppl. Figure 2A). Consistent with our flow cytometry data, the LPS-induced increase of ROS was abolished in the presence of GKY25, in contrast to the effects of the control peptide IVE25 (Fig. 2C and Suppl. Figure 2A). A dose-dependent reduction of ROS was also observed with increasing GKY25 concentrations (Fig. 2D). In addition, we assessed ROS generation by PMNs pre-treated with either IVE25 or GKY25. Also here, we observed a reduction of ROS levels in GKY25 pre-treated PMNs, when compared to the cells pre-treated with IVE25 (Fig. 2E). Taken together, these results demonstrate that GKY25 inhibits LPS-induced PMN activation.

### GKY25 induces specific CD62L shedding without activating PMNs

**Figure 3:**
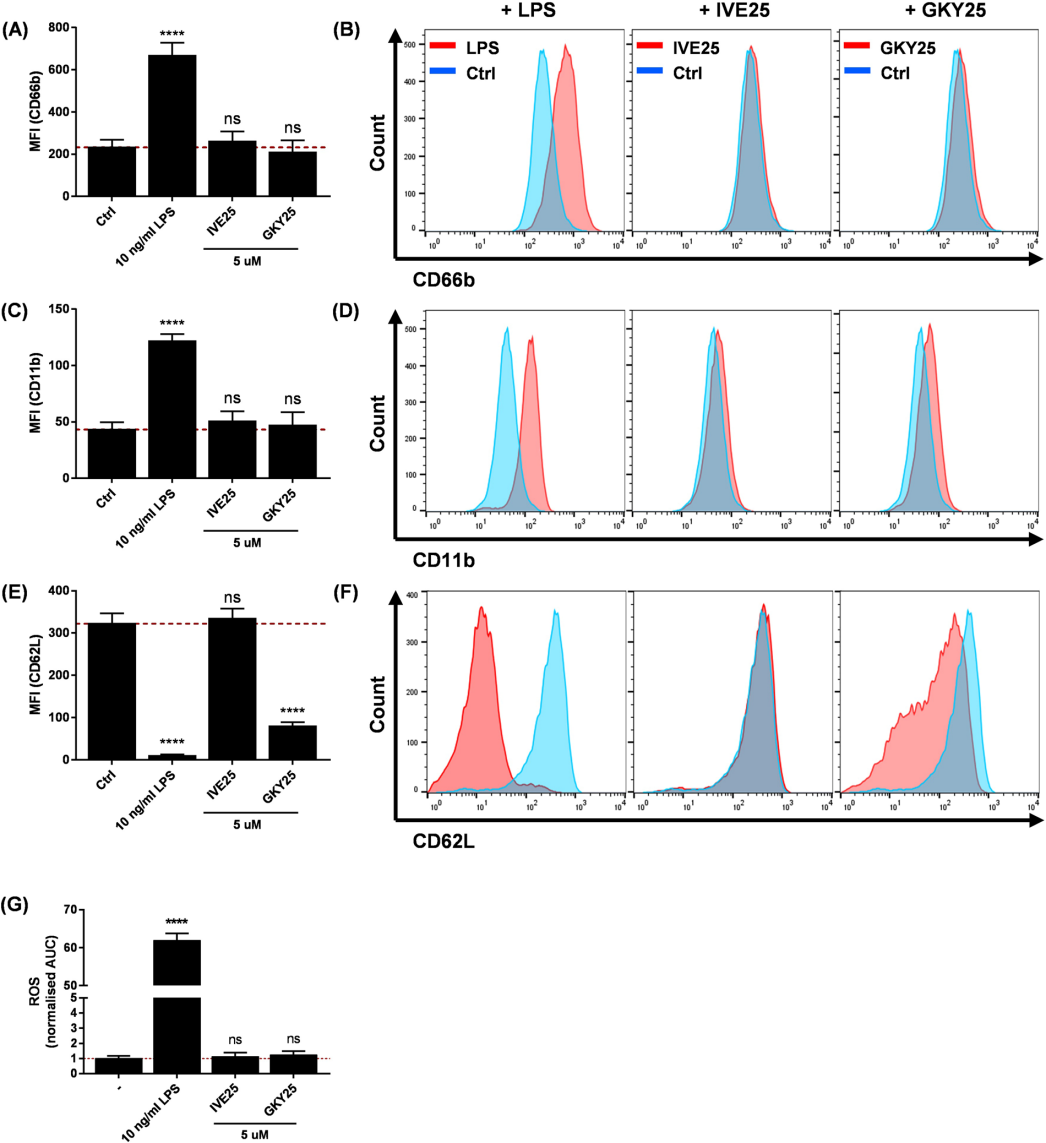
GKY25 induces CD62L shedding and modulates PMNs’ ROS response against LPS. PMNs treated with 10 ng/ml LPS, 5 µM IVE25 or 5 µM GKY25 for 1 hr at 37 °C were assessed by flow cytometry for surface expression of (**A,B**) CD66b, (**C,D**) CD11b and (**E,F**) CD62L (n = 3). Figures (**B**), (**D**) and (**F**) are representative histograms for the respective surface markers. (**G**) Pre-warmed PMNs (5 × 10^5^ cells/ml) were subjected to ROS assessment with 10 ng/ml LPS, 5 µM IVE25 or 5 µM GKY25 (n = 5). (−) Untreated baseline. One-way ANOVA. Figures are representative of 3 independent experiments.

**Figure 4:**
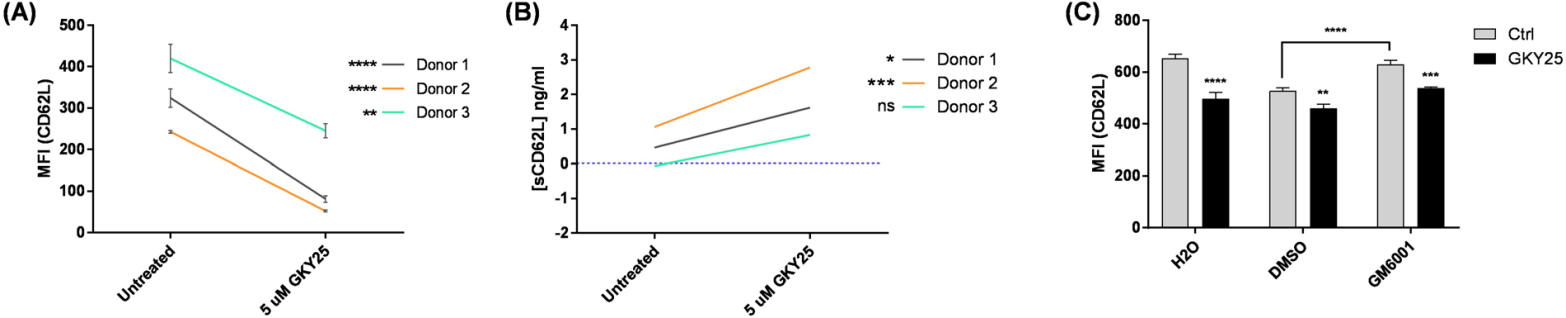
GKY25-induced CD62L shedding in presence of GM6001. PMNs (5 × 10^5^ cells/ml) isolated from 3 individuals were treated with 5 μM GKY25 for 1 hr at 37 °C. The cells were subjected to (**A**) flow cytometry to assess cell surface CD62L (mean ± SD of triplicate from each individual), whereas (**B**) the supernatants were used for ELISA to assess sCD62L (mean of technical duplicate from each individual). Multiple T-tests. (**C**) PMNs (1 × 10^6^ cells/ml) were also pre-incubated with 1:25 ratio by volume of H_2_O, DMSO or 100 μM GM6001 for 30 min at 37 °C before incubation with 5 µM GKY25 at the cell density of 5 × 10^5^ cells/ml for another 1 hr at 37 °C. Flow cytometry was performed to assess CD62L levels (n = 3). (−) Untreated baseline. Two-way ANOVA. Figure 4C is representative of 2 independent experiments.

Interestingly, we observed considerable CD62L down-regulation from PMNs co-treated with LPS and GKY25 (Fig. 2A,B) despite no upregulation in CD66b and CD11b, suggesting that GKY25 could induce the down-regulation of CD62L without activating PMNs. To this end, we investigated whether GKY25 has any direct, LPS-independent effects on PMN functions. First, we assessed PMN activation markers for early indication of any modulatory effects. Here, neither GKY25 nor IVE25 had any significant effects on the surface expression of CD66b (Fig. 3A,B) and CD11b (Fig. 3C,D); in contrast to the control peptide however, GKY25 induced significant down-regulation of CD62L (Fig. 3E,F). Surprisingly, while this event indicated PMN activation, no direct ROS response was detected against either GKY25 or IVE25 (Fig. 3G). Next, we compared the CD62L profile of PMNs isolated from the blood of 3 individuals. Here, the PMNs had reduced CD62L surface expression when treated with GKY25 (Fig. 4A) and these were matched with increased sCD62L levels in the respective supernatants, showing that the surface marker was shed (Fig. 4B). Similarly, the other neutrophil weak adhesion surface molecule, PSGL1, was also significantly reduced (Suppl. Figure 3A).

As the ectodomain shedding of CD62L can be mediated by leukocyte-associated metalloproteinases such as a disintegrin and metalloproteinase (ADAM)-8 and ADAM-17^25–27^, we next investigated whether the GKY25-mediated CD62L shedding was metalloproteinase-dependent. PMNs were pre-treated with a broad-spectrum metalloproteinase inhibitor, GM6001 before being subjected to GKY25 treatment. GKY25 mediated the down-regulation of CD62L also in GM6001 pre-treated PMNs (Fig. 4C). This suggests that GKY25 may trigger an alternative pathway yielding CD62L shedding.

### GKY25-induced shedding of CD62L affects PMN rolling *in vitro*

**Figure 5:**
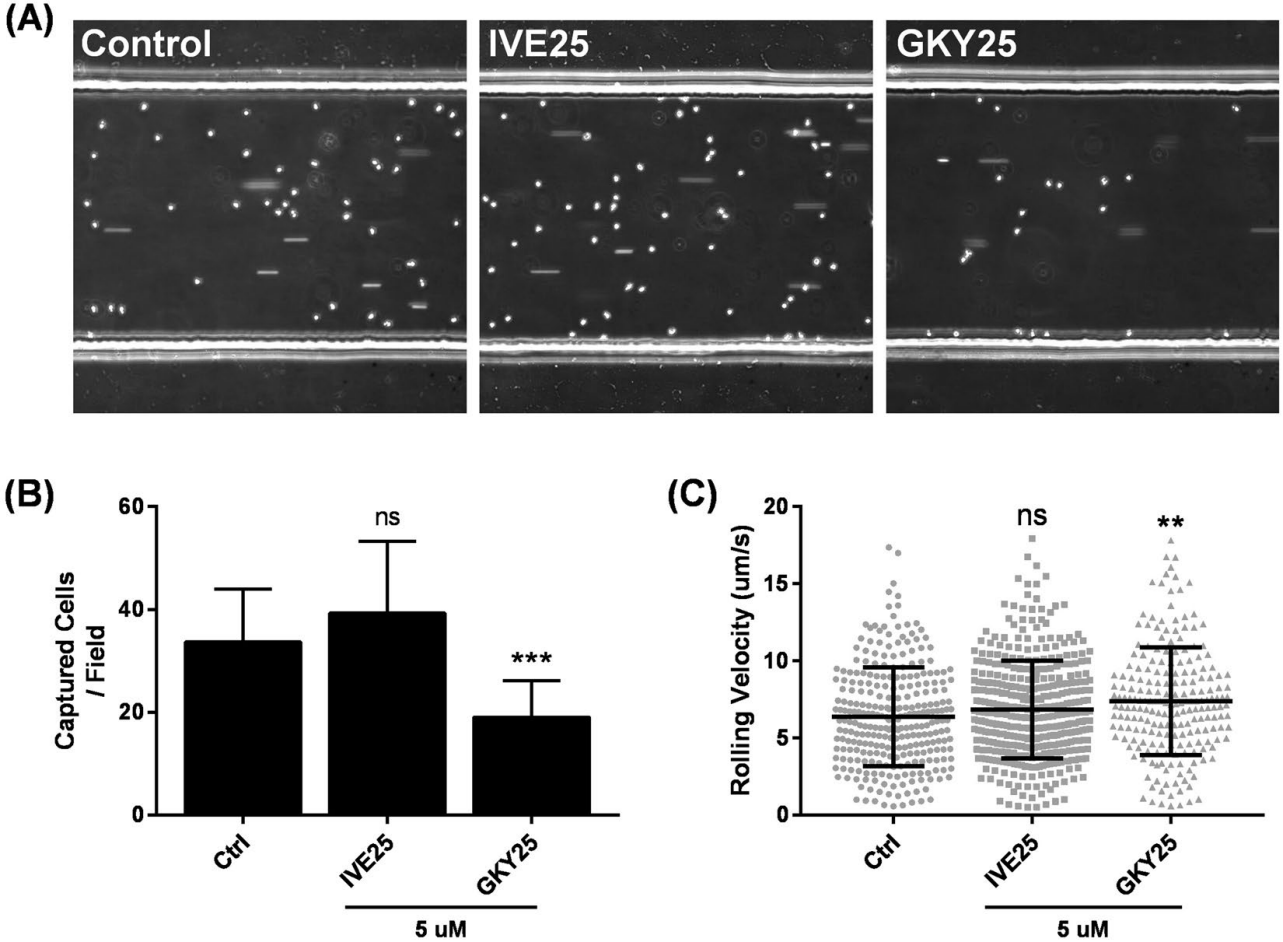
GKY25 attenuates neutrophil attachment and rolling *in vitro*. PMNs (1 × 10^6^ cells/ml) were treated with 5 µM IVE25 or GKY25 before being subjected to microfluidics flow assessment. (**A**) Representative images of attached cells/field. (**B**) Number of attached cells/field was counted from the images and (**C**) rolling velocities were determined from individual cells over the duration of time-lapsed video. One-way ANOVA. Figures are representative of 3 independent experiments.

CD62L is an important adhesion molecule which facilitates PMNs’ tethering and sensing along vascular walls^20, 25^. Since GKY25 induced PMN shedding of CD62L (Fig. 4A,B), we aimed to investigate whether this can impact PMN rolling *in vitro*. Rolling behaviour of PMNs treated with IVE25 or GKY25 was characterised using a microfluidics chamber coated with E-selectin, an adhesion molecule upregulated during endothelial inflammation and known to bind to CD62L^26, 27^. Under physiological flow conditions (~2 dyne/cm^2^), GKY25-treated PMNs demonstrated a lower capture rate on the E-selectin-coated surface versus untreated or IVE25-treated PMNs (Fig. 5A,B). Furthermore, GKY25-treated PMNs had significantly higher mean rolling velocities as compared to untreated or IVE25-treated PMNs (Fig. 5C and Suppl. Figure 4A,B). These results indicate that the recruitment of GKY25-treated PMNs during vascular inflammation may be impaired with weaker cell-surface attachment and higher rolling velocities possibly through the reduction of CD62L and PSGL-1 interactions with E-selectin.

### GKY25 reduces PMN chemotactic responses against IL8 in a CXCR1/2-independent manner

**Figure 6:**
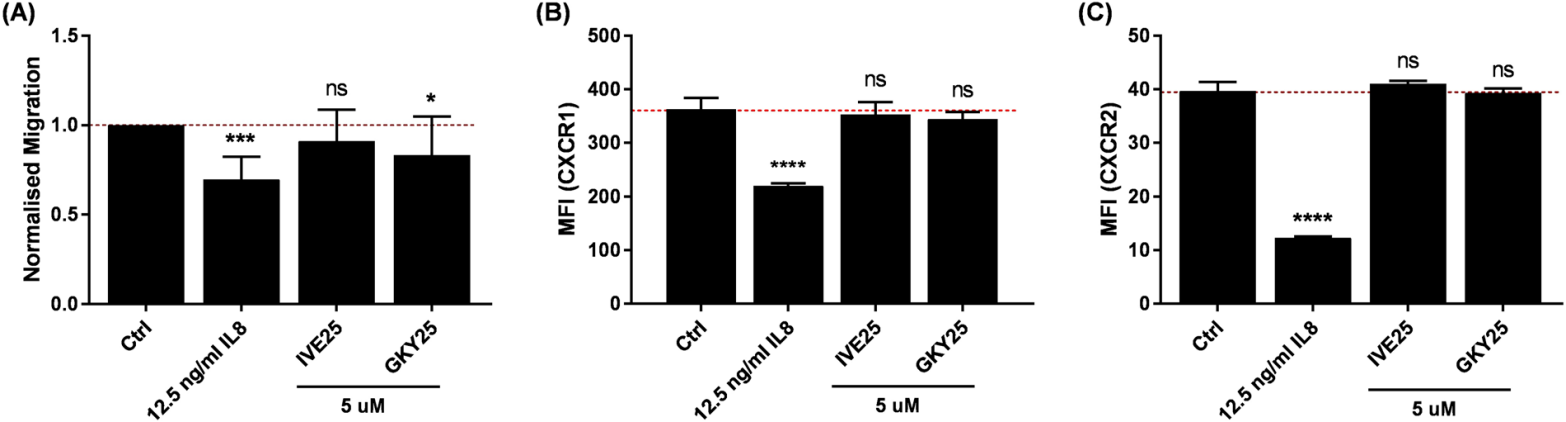
GKY25 reduces PMN chemotaxis. PMNs (5 × 10^5^ cells/ml) were pre-treated with 12.5 ng/ml IL8, 5 µM IVE25 or 5 µM GKY25 for 1 hr at 37 °C, washed, then subjected to (**A**) transwell migration against 12.5 ng/ml IL8 for 2 hrs at 37 °C (pooled normalised data from n = 11 independent experiments; One-way ANOVA with Dunnett’s test for multiple comparison) or flow cytometry assessment for (**B**) CXCR1 and (**C**) CXCR2; (−) untreated baseline, One-way ANOVA. Figures are representative of 2 (**B**) or 3 (**C**) independent experiments.

Migration across endothelial walls is an important process during neutrophil recruitment. To further investigate the potential effects of GKY25 on neutrophil migration, we assessed peptide-treated PMNs’ chemotactic response against the chemokine IL8. As expected, PMNs pre-treated with IL8 significantly impaired chemotaxis against IL8 as compared to untreated or IVE25 pre-treated PMNs (Fig. 6A). Similarly, GKY25 pre-treated PMNs showed a significantly reduced chemotactic response (Fig. 6A). As IL8 acts through the receptors CXCR1 and CXCR2 and HDPs are reported to act through G-protein coupled receptors (GPCRs; mostly chemokine receptors)^13, 28, 29^, we also assessed receptor expressions on GKY25-treated PMNs. Interestingly, while IL8 induced surface downregulation of CXCR1 and CXCR2, GKY25 did not (Fig. 6B,C). This suggests that GKY25 may affect other pathways affecting PMN chemotaxis, possibly downstream of chemokine receptor activation.

### GKY25 ameliorates LPS-induced local ROS production *in vivo*

**Figure 7:**
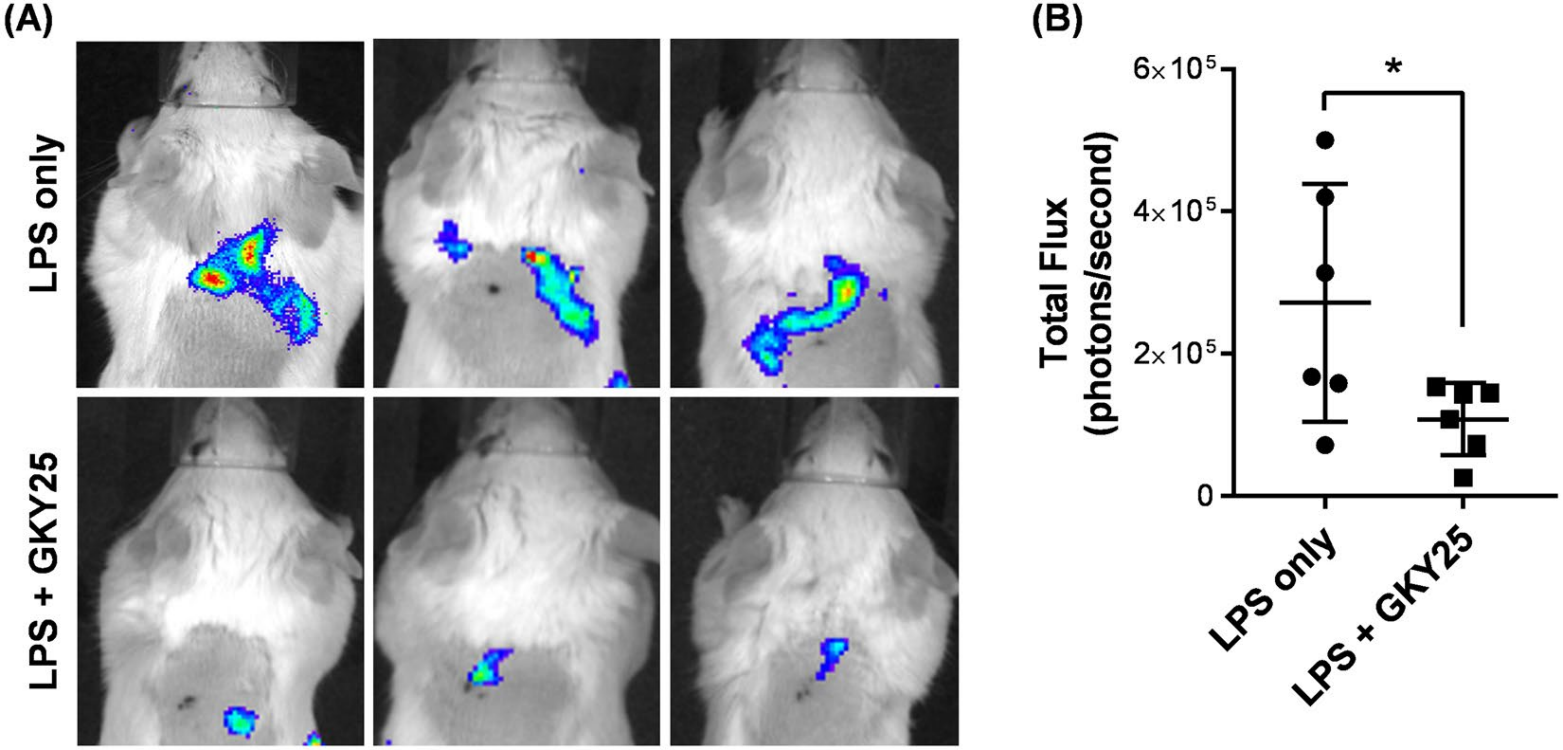
GKY25 ameliorates LPS-induced local ROS production *in vivo*. (**A**) Representative *in vivo* imaging of ROS with L-012 in mice. Using an IVIS SPECTRUM 200 Imaging System, ROS production was imaged and measured in the scruff of the neck. Mice were pretreated with GKY25 followed by subcutaneous LPS administration. Luminescent signal was detected 10 min after L-012 injection. (**B**) The statistical significance was calculated between sham- and GKY25-treated mice (n = 6 mice). Unpaired T-test.

As neutrophils are the first cells to respond to danger signals, we were interested to translate our *in vitro* findings on neutrophil responses to a relevant *in vivo* situation. Here, we sought to assess local ROS production induced by LPS as a measure of neutrophil response. LPS was injected subcutaneously into the dorsal region of untreated or GKY25-treated mice before ROS measurement. As observed, LPS treatment resulted in a significant increase in ROS production, localised to the site of injection (Fig. 7A upper panel and 7B). In contrast, significantly reduced ROS production was observed in GKY25-treated mice (Fig. 7A lower panel and 7B), showing that GKY25 administered intraperitoneally was able to inhibit LPS-induced neutrophil responses also *in vivo*. In addition, to explore the effects of GKY25 more in detail, the peptide was administered either as a pre-treatment given intraperitoneally before LPS administration subcutaneously (as above) or co-administered with LPS subcutaneously. Skin tissues were then harvested and immuno-stained with anti-neutrophil antibodies (NIMP-R14) to assess neutrophil localisation in either condition. We observed marked neutrophil infiltration in the extravascular space in response to LPS which was only reduced in the setup when GKY25 was given subcutaneously (Suppl. Figure 5A,B) and not as pre-treatment intraperitoneally (Suppl. Figure 5C,D). Taken together, GKY25 can reduce neutrophil activation by either direct neutrophil modulation or LPS scavenging, leading to inhibition of LPS-induced neutrophil responses.

## Discussion

In this study, we demonstrate for the first time the immunomodulatory effects of GKY25 on neutrophils. First, we show that GKY25 can inhibit LPS-induced neutrophil activation. In addition, GKY25 can modulate neutrophils independently by inducing the shedding of CD62L, thus affecting neutrophil attachment and rolling *in vitro*. Second, GKY25 impaired neutrophil chemotactic response against IL8 independent from CXCR1 and CXCR2 regulation. Finally, mice treated with GKY25 showed significant reduction in local LPS-induced ROS production, indicating that the peptide can also modulate neutrophil immune responses *in vivo*.

Neutrophil tethering and sensing along endothelial walls are all important processes mediated by weak adhesion molecules such as CD62L and PSGL1 during homeostasis and inflammation. Notably, GKY25 induced both adhesion molecules’ shedding on neutrophil surface without the concomitant upregulation of CD66b and CD11b that would otherwise indicate neutrophil activation^19, 30^ (Figs 3A–0F, 4A,B and Suppl. Figure 3). Consequently, GKY25-treated PMNs presented reduced initial attachment and increased rolling velocities on E-selectin-coated surfaces (Fig. 5), presumably due to reduced surface engagement of CD62L and PSGL1 to E-selectin. Hypothetically, the shed CD62L and PSGL1 may have played synergistic roles in binding to E-selectin, thus reducing the total available ligand for PMN attachment. Furthermore, GKY25-treated PMNs displayed a reduced chemotactic response against IL8 *in vitro* (Fig. 6A). This potential modulation on neutrophil recruitment could play important roles in host defence; early studies by Jutila *et al*. (1989) demonstrate that the use of monoclonal antibodies against CD62L reduced neutrophil recruitment to inflamed peritoneum in mice^31^. Mice deficient in CD62L were later shown to have reduced immune infiltration into inflamed peritoneum and induced hypersensitivity in mouse ears^32^. On the contrary, when CD62L was protected from endoproteolytic cleavage, sustained and increased neutrophil recruitment was observed in inflamed peritoneum^33^. Notably, CD62L which primarily acts as a weak adhesion molecule, has been implicated to hinder CD62L-deficient neutrophil locomotion *in vivo* ^34^. In addition, a study has shown that human neutrophils with reduced CD62L expression exhibited reduced chemotactic response to fMLP^35^. Indeed, ligation of CD62L on leukocyte surfaces (including neutrophils) has been reported to induce signal transduction and activate integrin function as well as enhancing lymphocytes’ chemotaxis^36–38^, hence suggesting that the lack of CD62L on neutrophil surface could also affect their chemotaxis and consequent recruitment. Similarly, PSGL1 has been shown to mediate neutrophil tethering both *in vitro* and *in vivo* ^39, 40^, further suggesting that GKY25-induced shedding of weak adhesion molecules may synergistically modulate neutrophil responses during host defence and inflammation.

The exact mechanism by which GKY25 induces shedding of these weak adhesion molecules and whether it directly modulates neutrophil response to IL8, remains however to be characterised. CD62L is anchored to calmodulin, which upon cellular activation, exposes CD62L’s ectodomain region, leading to cleavage by several metalloproteinases, most notably by the enzyme ADAM17^25, 26, 41, 42^. Interestingly, GKY25 induced CD62L shedding in the presence of the broad-spectrum metalloproteinase inhibitor GM6001 (Fig. 4C). In this context, it should be noted that studies have shown that CD62L can indeed be shed via other mechanisms which are protease-independent, such as mechanical stress under flow conditions^43^, after cross-linking with anti-CD62L antibodies or CD62L-specific compounds^44, 45^, during apoptosis^26^ and in response to microparticle generation^46, 47^. Thus, it is possible that GKY25 has other molecular targets related to these pathways.

Integral to our study is the demonstration on GKY25’s immunomodulatory effects *in vivo* (Fig. 7 and Suppl. Figure 5). Since the results indicated that GKY25 modulates neutrophil functions *in vitro* (Figs 2–6), we aimed to study the peptide’s *in vivo* effects. For this purpose, GKY25 was injected intraperitoneally followed by LPS injection subcutaneously before ROS was measured. In this context, it should be mentioned that LPS has been reported to exert its major effects in priming of neutrophils, facilitating activation by substances such as N-formyl-methionyl-leucyl-phenylalanine (fMLP)^21–23, 48^. Hence, it may be argued that LPS, at physiologically relevant concentrations, is not a strong activator of the respiratory burst. However, LPS has also has been reported *per se* to induce release of ROS in human PMNs *in vitro* ^21–23^ as well as *ex vivo* in whole blood^49, 50^, albeit at lower levels relative to those observed after treatment with strong activators, such as PMA^22, 48^ (see also Fig. 2C and Suppl. Figure 2A). Furthermore, LPS alone can activate interleukin-1 receptor-associated kinase-4 (IRAK-4) phosphorylation of p47^*ph*^ *°* ^*x*^, a subunit of the NADPH oxidase complex responsible for ROS production in neutrophils^24^. Indeed, IRAK-4-deficient neutrophils from patients cannot produce ROS in response to LPS^22^. Thus, consistent with previously published results on LPS-induced ROS generation^51, 52^, we observed an increase in ROS after subcutaneous injection of LPS in the experimental model used. The ROS response was reduced significantly in GKY25-treated mice (Fig. 7), suggesting that neutrophils were either not infiltrating, or not being activated at the site of LPS injection. Analyses of skin sections from an identical experiment showed that neutrophil levels were unaffected (Suppl. Figure 5C,D), indicating that the translation of the peptide effects on chemotaxis *in vitro*, as observed in Figs 5 and 6, to the *in vivo* situation needs further studies. Nevertheless, the *in vivo* data are compatible with the reduction of LPS-induced activation as observed *in vitro* (Fig. 2E).

Neutrophil infiltration was however reduced in the subcutaneous model when LPS and GKY25 were co-administered (Suppl. Figure 5A,B), which is compatible with direct LPS blocking, thus leading to less LPS-induced neutrophil recruitment. This finding is consistent with previous reports where LPS and GKY25 were sequentially injected into the peritoneal space, leading to a reduction of inflammation and increase in overall survival in mouse models of endotoxin-shock^10^. Notably, GKY25 significantly reduced cytokine levels, including tumour necrosis factor alpha (TNF-α), in such animal models^10^. Of relevance from an *in vivo* perspective is that TNF-α has been reported to sensitise PMNs to LPS, yielding ROS responses to LPS levels of 1 ng/ml, doses 10$\mbox{--}$100 times lower than those needed for stimulation with LPS alone^53^. It is therefore possible, that an overall reduction of cytokines by treatment with GKY25 may contribute to the observed inhibitory effects of the peptide on LPS-induced PMN activation *in vivo*. Taken together, the demonstration that PMNs pre-treated with GKY25 showed reduced ROS response against LPS *in vitro* (Fig. 2E) and *in vivo* (Fig. 7 and Suppl. Figure 5) indicate that GKY25 affects neutrophil responses at multiple levels. Of note is that both setups could potentially be translated to clinical therapies. For example, in certain surgical situations where endotoxin is at risk of being spread, pre-surgical administration of GKY25 could hypothetically show beneficial effects.

Taken together, this study demonstrates a novel mechanism by which GKY25 affects neutrophil functions by attenuating their rolling and migration through the shedding of weak adhesion molecules such as CD62L *in vitro*. GKY25 may also modulate neutrophils *in vivo*, hence down-regulate immune responses. Future studies are clearly needed to understand the exact mode of action of GKY25. Nevertheless, this study shows that GKY25 can be an interesting candidate for the development of novel therapeutics against inflammatory and infective conditions.

## Electronic supplementary material

Supplementary Information
