# Supplementary material for: Thrombin-derived host defence peptide modulates neutrophil rolling and migration in vitro and functional response in vivo
Source: Sci Rep. 2017 Sep 11;7:11201. doi: 10.1038/s41598-017-11464-x (PMC5593972; doi:10.1038/s41598-017-11464-x)

Thrombin-derived host defence peptide modulates neutrophil rolling and migration *in vitro* and functional response *in vivo*

#Chun Hwee LIM[1,2], Manoj PUTHIA[3], Marta BUTRYM[3], Hui Min TAY[1], Michelle Zi Yi LEE[1,4], Han Wei HOU[1] and Artur SCHMIDTCHEN[1,3]

[1]Lee Kong Chian School of Medicine, Nanyang Technological University, Singapore

[2]Nanyang Institute of Technology, Health and Medicine, Interdisciplinary Graduate School, Nanyang Technological University, Singapore

[3]Division of Dermatology and Venereology, Department of Clinical Sciences, Lund University, Lund, Sweden

[4]School of Chemical and Life Sciences, Singapore Polytechnic, Singapore

#Corresponding author: Chun Hwee Lim, Lee Kong Chian School of Medicine, Nanyang Technological University, email: limc0186@e.ntu.edu.sg

Supplementary Information

Supplementary Figure 1: Effects of GKY25 on PMN toxicity and functional ROS response. (A) PMNs (5 X 10^5 cells/ml) were treated with either IVE25 or GKY25 for up to 4 hrs at 37°C before LDH activity measurement (n = 3). **(B)** PMNs (1 X 10^6 cells/ml) were pre-treated with either LPS, IVE25 or GKY25 for 1hr at 37°C before being subjected to 25 nM PMA stimulation for ROS response (n = 5). One-way ANOVA. Figures are representative of 2 independent experiments.

Supplementary Figure 2: Assessment of GKY25 inhibition of LPS-induced ROS generation by flow cytometry. PMNs (1 X 10^6 cells/ml) pre-incubated with 10 μM H_2DCFDA for 30 min at 37°C were treated with 25 nM PMA for 15 min as positive control or co-treated with 5 μM IVE25 or 5 μM GKY25 with 10 ng/ml LPS for 1 hr at 37°C before flow cytometry assessment for **(A)** H_2DCFDA, **(B)** CD11b or **(C)** CD62L. (---) Untreated baseline. One-way ANOVA. Figures are representative of 2 independent experiments.

Supplementary Figure 3: Modulation of PSGL1 by GKY25. PMNs (5 X 10^5 cells/ml) isolated from 3 individuals were treated with 5 μM GKY25 for 1 hr at 37°C before flow cytometry. Multiple T-tests.

Supplementary Figure 4: Rolling frequencies of PMNs on E-selectin coated PDMS microdevice. PMNs (1 X 10^6 cells/ml) were treated with 5 μM IVE25 or GKY25 before being subjected to microfluidics flow assessment. Rolling frequencies of GKY25-treated PMNs were

plotted with **(A)** untreated PMNs and **(B)** IVE25-treated PMNs. Figures were representative of 3 independent experiments.

Supplementary Figure 5: Neutrophil infiltration *in vivo*. (A-B) Mice were administered with LPS in the absence or presence of GKY25 before being sacrificed after 1 hr (n = 4). **(C-D)** Mice were also pre-treated with GKY25 intraperitoneally before LPS administration subcutaneously (n = 3). **(A, C)** Skin sections were stained with anti-neutrophil antibody (NIMP-R14, red) and DAPI (blue) and **(B, D)** used for scoring for neutrophil infiltration. Unpaired T-test.

Supplementary Figure 1

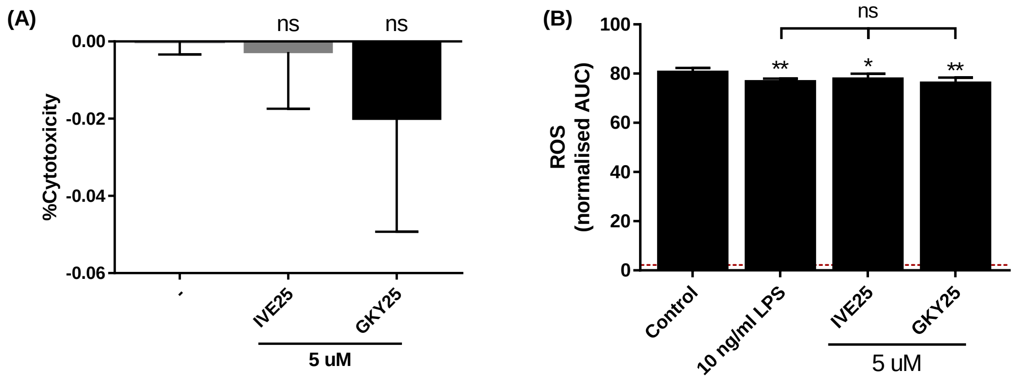

Supplementary Figure 2

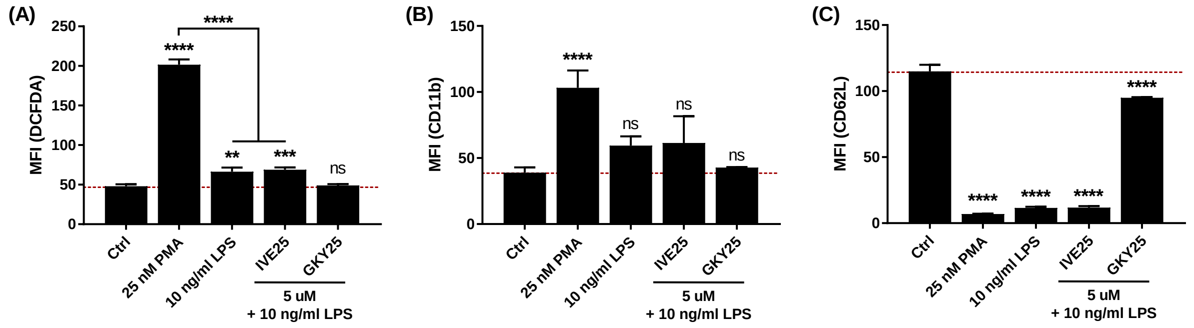

Supplementary Figure 3

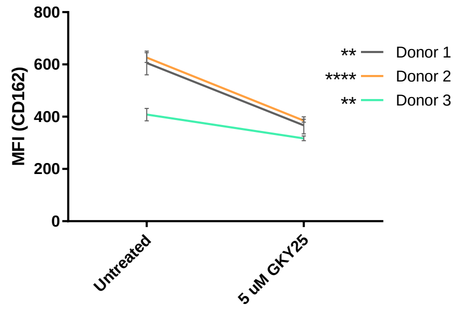

Supplementary Figure 4

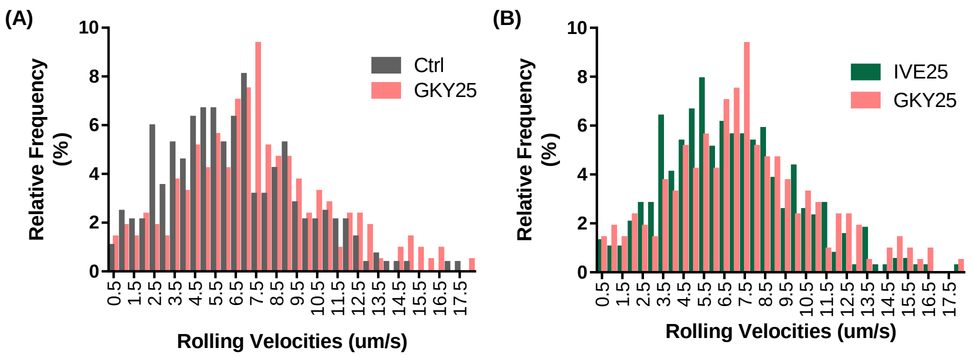

Supplementary Figure 5

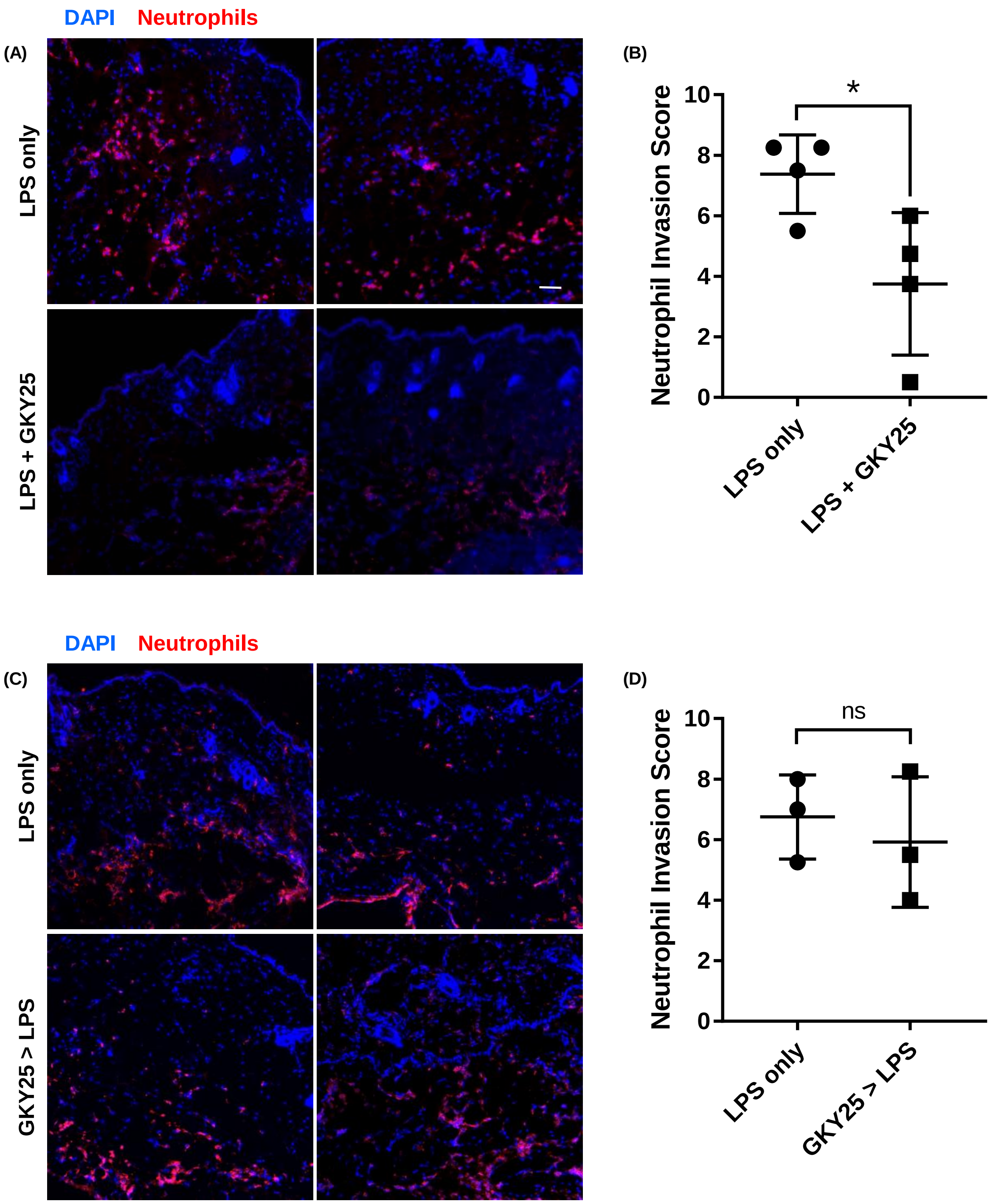

Supplement: Supplementary file 1 — Supplementary Information [file 41598_2017_11464_MOESM1_ESM.pdf]
